# From 'omics' to complex disease: a systems biology approach to gene-environment interactions in cancer

**DOI:** 10.1186/1475-2867-10-11

**Published:** 2010-04-26

**Authors:** Sarah S Knox

**Affiliations:** 1 Program in Clinical and Population Epigenetics, Dept. of Community Medicine West Virginia University School of Medicine, PO Box 9190, Health Science South Morgantown, WV 26506, USA

## Abstract

**Background:**

Cancer is a complex disease that involves a sequence of gene-environment interactions in a progressive process that cannot occur without dysfunction in multiple systems, including DNA repair, apoptotic and immune functions. Epigenetic mechanisms, responding to numerous internal and external cues in a dynamic ongoing exchange, play a key role in mediating environmental influences on gene expression and tumor development.

**Hypothesis:**

The hypothesis put forth in this paper addresses the limited success of treatment outcomes in clinical oncology. It states that improvement in treatment efficacy requires a new paradigm that focuses on reversing systemic dysfunction and tailoring treatments to specific stages in the process. It requires moving from a reductionist framework of seeking to destroy aberrant cells and pathways to a transdisciplinary systems biology approach aimed at reversing multiple levels of dysfunction.

**Conclusion:**

Because there are many biological pathways and multiple epigenetic influences working simultaneously in the expression of cancer phenotypes, studying individual components in isolation does not allow an adequate understanding of phenotypic expression. A systems biology approach using new modeling techniques and nonlinear mathematics is needed to investigate gene-environment interactions and improve treatment efficacy. A broader array of study designs will also be required, including prospective molecular epidemiology, immune competent animal models and *in vitro/in vivo *translational research that more accurately reflects the complex process of tumor initiation and progression.

## Introduction

Large population-based studies have provided important information concerning trends in morbidity and mortality, and have helped identify genotypes, behaviors, and environmental factors associated with multiple chronic diseases. Based on this knowledge, it has become increasingly evident that the chronic diseases responsible for the greatest mortality, e.g., cardiovascular disease and cancer, occur in a context of interaction between multiple genes, environmental risk factors and epigenetic changes. Although the complexity of causal factors associated with these diseases has been known for some time, our understanding of gene-environment interactions has not kept pace. For a long time it was believed that the relationship between genes and environmental factors was essentially additive, i.e., that genotypes explained a certain fixed amount of the population variance in disease prevalence and that when environmental factors were added to genotype, heredity and environment summed to 100 percent.

Interactions were defined *statistically*. In the simplified example below (equation adopted from Pedersen NL, [1]) the phenotype of Twin A is predicted from the phenotype of Twin B plus the environment, plus the interaction of these 2 main effects.

The problem with this approach is that linear equations do not accurately reflect the complexity of nonlinear interactions at a molecular level. Gene expression and function can vary based on the surrounding micro environment, which varies in response to multiple internal and external cues. The mechanisms responsible for these variations in response to the surrounding environment are the epigenome in conjunction with *cis*-regulating mechanisms. DNA is analogous to computer hardware and the software needed to program it is the epigenome [2]. Cells throughout the body are for the most part, genetically identical. What differentiates one tissue or organ from another is gene expression. Just as gene expression varies between organs, it can also respond and adapt to multiple environmental cues - dietary nutrients, smoking, other toxicant exposure, and psychosocial stress.

Most environmental factors that precipitate epigenetic changes affect *multiple systems simultaneously*. Thus, the overall effect depends on how these functional changes interact. Two different exposures (e.g., diet and smoking) may have influences on the gene expression of a single polymorphism that are mutually antagonistic or synergistic. The ultimate expression of that allele will depend on the combined effect including enzymes, transcription factors, genes, and signaling pathways. Thus, the 'genetics' component of the phenotype is neither static nor additive. Gene expression is dynamic. In addition to the complex biology of genotype - environment interactions, epistatic (gene × gene) interactions, are also environmentally influenced via the epigenome. This means that a single gene can have different functional responses, depending on its ambient surroundings. A good illustration is the P53 tumor suppressor gene. P53, which involves complex signaling pathways central to tumor development, responds to a broad range of signals that determine whether the cellular response will be DNA repair, cell metabolism, autophagy, apoptosis or cell cycle arrest [3,4].

This explains why genes and environment cannot be understood with linear statistical equations. The effects of the environment on phenotype are much too complex to be adequately accounted for in an additive model. It also clarifies how the environment can interact with genotype without there being a statistical 'main' effect for either genotype or environment. The intertwining of genetics, epigenetics and environmental factors is one of nonlinear dynamically interacting systems.

These interactions have major implications for study design and data analysis. The term complexity applies not just to the number of causal exposures (e.g., smoking, diet, toxicity, genotype) but to the multiplicity of integrated systems that interact in response to these exposures. Using a statistical model that first examines the significant (main) effects of individual factors and next interactions between two main effects, would be inappropriate where several factors not having 'main' effects are working synergistically, but not additively, to achieve a result that none of them could achieve alone.

Nonlinear dynamical interactions are also highly relevant for the design of genome wide association studies (GWAS), which examine associations between a genotype and phenotype in populations after controlling for relevant confounders. The design of these studies does not take into consideration interactions that allow the same genotype to alter function in different contexts, leading to multiple phenotypes being associated with the same genotype. The naïve assumption of GWAS design is that, regardless of context, a genotype will be associated with the same phenotype. The failure to replicate in many candidate gene studies [5,6] is poorly understood but has variously been attributed to factors such as epistasis, population drift, population stratification, and genetic diversity. These are all important factors. However, the multiple nonlinear influences on gene expression are the primary culprit limiting the usefulness of GWAS design for investigating causal influences of genotype on phenotype. Rather than assuming that the candidate gene is not relevant because the results are not significant, we should be asking whether there are particular circumstances under which the allele in question explains a significant amount of the variance and others where it does not.

New methodologies and mathematics are being developed to generate predictions about interactions at multiple levels of complexity, and to test these predictions against experimental data so that the accuracy of predictions is continually refined. The focus of this article will be on the role of systemic malfunction in the etiology of cancer, and the necessity of finding new approaches to clinical oncology.

## Biological Complexity

### Complexity in Dynamical Systems

The 'whole', i.e., the phenotype or disease condition that we are trying to treat and prevent, is greater than the sum of its parts. This is why complex diseases cannot be understood by studying individual genes, signaling pathways, behaviors or environmental exposures without observing them in a functional context. The emergence of new properties evolves as the level of complexity increases: from molecules to proteins, from proteins to tissues and so on. Hemoglobin is a good example. The part of the blood that transports oxygen, is composed of four hemoproteins [7] which do not have the ability to transport oxygen. However, when they combine, some of their original properties and functions, such as hydration patterns, are lost. What emerges is a property that the individual molecules did not have, namely the ability to transport oxygen. This concept is important because it is a good analogy for complex diseases, which involve many gene-gene and gene-environment interactions that create emergent properties not present in the individual components.

Cells and organisms are also dissipative because they are open systems that continually exchange energy and matter with their surrounding environments [8]. This exchange, occurring under ever varying conditions, creates a dynamic in the body's biochemistry. An example of how the state of flux plays out at the molecular level can be illustrated by *conformer *molecules. These molecules can assume different 3-dimensional spatial arrangements without breaking their chemical bonds by having parts of the molecule simply rotate around a single bond. The shape of a conformer can be influenced by its surroundings. When in water, it will hide its hydrophobic side-chains in a core and expose its polar groups to the solvent. Their ability to fluctuate in this manner is dependent not only on the environment but on their chemical composition (e.g., their chemical bonds). The number of possible geometric forms that a molecule can assume is called the *conformational space *[7], which constitutes the expression of different characteristic states or properties of the molecule. "The fluctuation of form and function generates a number of molecular states, which are snapshots of the molecule at a given point in time" [7]. The range of possible properties is called the *property space*. At each level of complexity the property space changes.

This flexibility serves an adaptability of function necessary for the dynamic equilibrium that contributes to the survival of the organism. The term 'dynamic equilibrium' is used instead of 'homeostasis' to illustrate that the state space is limited but not static in healthy individuals (e.g., heart rate stays within in a limited range but varies greatly over time). This also applies to the DNA strand (the double helix of base pairs), which is wound around 8 histone proteins and packed into nucleosomes that have the ability to control access to the DNA by polymerases and transcription factors. This efficient packaging fits the DNA into the nucleus and controls when and in what context the gene will be expressed. However, there is an ongoing exchange between the nucleus, the cell and the extra cellular environment that influences the shape of the nucleosome, promoters, transcription factors and other molecules. Thus, the nucleosomes are dynamic and can undergo conformational fluctuations in the temporal range of seconds to microseconds [9]. A molecule's state at any given time is not just determined by the individual properties of its subcomponents but by the system as a whole. (For a more complete discussion of the dynamics of living states, please see Agutter & Wheatley [10].

This means that when we do an *in vitro *experiment and expose a gene, receptor or signaling pathway to a specific substance, what we are observing is how that molecule behaves when exposed to that specific substance *in that particular context *(e.g., *in vitro*, or *in vivo *in Drosophila or an immune-compromised mouse). If we are using an animal model, it is highly likely that the reaction will vary depending on how well the immune system functions and on the mode of exposure (e.g., intraperitoneal injection, inhalation). The transition from how a rodent responds to how a human responds is another large leap. We must remember that even when a rodent gene is very similar to that of humans, the mouse itself is very different [11]. This does not reduce the importance of *in vitro *or animal model experiments, but requires caution in how we interpret them. An individual gene, receptor or signaling pathway may be a marker of disease progression or increase our ability to predict outcome at a certain stage of disease, and yet not lead to an understanding of how this factor interacts systemically with respect to host susceptibility or etiology. If we don't understand etiology, then our treatment methods may be focusing on the wrong targets. The wheels are significantly associated with the movement of a car uphill but do not explain the car's ability to accelerate. Without a motor, the wheels, would in fact, only revolve in a downhill direction. So, although wheels are an integral aspect of a car's structure and function, examining a wheel in isolation does not provide sufficient information to help us understand what enables a car to accelerate. To understand that, we need more information about how the wheels, motor, transmission and fuel systems work together. In other words, we need knowledge about the integrated function of the whole system.

To summarize, confining research to a particular level, be it the molecular, tissue, animal, or population, is not sufficient for unraveling complex diseases in humans. It is becoming increasingly evident that only transdisciplinary research with a broader range of study designs and new methods that facilitate the understanding of nonlinear systems will be able to accomplish this. Mathematical tools continue to be developed to grapple with the problem of modeling and understanding the complexity of these dynamic interactions.

### Nonlinear Dynamics

The term *nonlinear dynamics *refers to changing conditions that do not occur in a proportional or linear manner. An example of a linear relationship would be a situation where every increase in the number of cigarette packs smoked is associated with a proportional decrease in months of survival time in the smoker. A nonlinear relationship can be exemplified by a drug that promotes health up to a certain dose, but which becomes toxic and can result in death above this threshold. The dynamics of chemical and biological systems are nonlinear, which has important implications for function because they exhibit varying spatiotemporal patterns depending on the health of the system as a whole. Most of the systems in the body, including brain waves, neuroendocrine secretions, heart rhythms, chemical processes, and epigenetic mechanisms are nonlinear and also dynamic. This allows them to fluctuate in response to the body's needs and varying environmental demands, to assure survival.

Nonlinear mathematical methods have been successful in explaining the dynamics of chemical and biological systems as well as outcomes in clinical medicine [12]. Even at the molecular level, it has been demonstrated that introns (noncoding DNA sequences) have remarkably long range correlations that can extend over thousands of base pairs, while coding sequences do not [13,14]. Clinically, nonlinear methods have improved prognostic ability in the fields of cardiology (e.g., predicting cardiomyopathy [12]), sudden death [15] and aging [15]. By enhancing statistical probability analyses with mathematical methods from physics, knowledge and interpretation of variation in multiple biological signals have been greatly enhanced. Through these advances, we have learned that the concept of homeostasis which was previously used to describe the desired state of bodily systems is a misnomer. Stasis is not a healthy condition at all. Health is maintained by a state of dynamic equilibrium which allows the body to adapt and respond while maintaining stability.

One of the areas of medicine that has been difficult to resolve with linear methods is the unpredictability of systems that suddenly shift from being clinically stable to being clinically unstable (e.g., asthma attacks, epileptic seizures). It is often the case that there is little visible indication that the transition is about to take place [16]. A recent review of these nonlinear transitions [16] described a condition of 'critical slowing down' that seems to be generic across systems. Before the critical transition points (known as 'catastrophic bifurcations') the system's ability to respond to small perturbations decreases, moving the system away from flexibility towards stasis. The critical slowing down begins long before the bifurcation point, so the ability to respond to small perturbations can be used as an indicator of how close the system is to a critical point. However, in clinical situations such as chronic asthma, it is usually impossible to continually test reactions to pre-determined stimuli. Therefore, a technique for monitoring the state of a biological system with mathematical signal analysis has been developed. The slowing down process in a system's responses indicates that the moment-to-moment signal fluctuations are becoming increasingly similar. Thus, autocorrelations in temporal patterns can be a reliable indicator of how close the system is to a critical shift. Similar techniques can also be adopted for spatial patterns.

### Systems Biology

Increasing awareness of the complex dynamics of living systems has led to a new field of research called systems biology. It evolved partly because of the need for computational methods that can deal with the nonlinearity in signaling pathways in relationships between genotype and phenotype. This need developed from the realization that genetic diversity does not account for the diversity of physiological functions, nor does *cis*-rgulatory control of DNA lead directly to an understanding of organism function [17]. The poor reproducibility of candidate gene studies in predicting phenotype and the failure to explain variability in phenotype by reducing systemic complexity to the properties of the subcomponents has led to the recognition that new approaches are required. Although systems biology tends to focus on molecular networks, it differs from traditional molecular biology in that it utilizes analytic techniques designed to account for emergent properties arising from the context, adaptability and plasticity of function [18] in signaling pathways that contribute to disease phenotypes and treatment responsiveness. It has been demonstrated that the use of nonlinear dynamical measures works equally as well for 'omics' data as for biological rhythms represented by the ECG and EEG [19]. Although systems biology has been defined in various ways, it usually includes the quantitative analysis of dynamic interactions among several components of a system (biological, chemical), with the intent of understanding the behavior of the system as a whole rather than the behavior of the individual components [20,21].

Rather than compartmentalizing individual risk factors (e.g., blood pressure or lipid concentrations in CVD) and treating them as if they were separate and independent, systems biology examines their interactions and the complexity of systemic response to treatments. The key concept is that a treatment intended for one particular symptom (e.g. tumor growth) affects multiple other systems; hence an understanding of these interactions is essential for treatment efficacy. If a treatment reduces tumor size but simultaneously reduces immunologic responsiveness, then it may inadvertently promote new tumor growth. This is why a systems biology approach is particularly useful with complex diseases that involve multiple organ systems and etiologic contributors. It would not be necessary or appropriate in a straightforward case of a bacterial infection in a young and otherwise healthy person where a simple antibiotic suffices. However, a similar infection in a septuagenarian with several co-morbidities who is being treated with multiple pharmacologic regimens presents a more complex clinical picture and would benefit from a more systems oriented approach.

Chronic conditions that develop over a long time course and have multiple genetic and environmental contributors cannot be properly understood without studying the dynamic spatial and temporal interactions of the affected systems. Although this paper focuses on cancer, the concepts apply to many other conditions (e.g., cardiovascular disease), as well. These chronic conditions could greatly benefit from a multidisciplinary systems biology approach. The utility of systems dynamics for understanding host susceptibility and the consequent implications for the design of research related to treatment is relevant to both.

## Cancer

### The Cancer Pharmacology Paradigm

Cancer pharmacology has been a difficult area of research. Despite tremendous effort and billions of dollars invested, only marginal improvements in treatment outcome have been achieved since the 'war on cancer' was first declared. A recent review concludes that oncology has one of the poorest records for investigational drugs in clinical development [22]. It is time we examined the reasons and planned a strategy for addressing them.

Part of the problem may actually be the frame of reference for pharmacologic models of cancer [23]. The current conceptualization of cancer as a morphological entity that must be attacked and eliminated has achieved only limited success [23]. This paradigm focuses on attacking cancer the way we attack and kill invading bacteria. The problem with this approach is that cancer cells are not alien invaders; they are part of our own bodies.

Drugs used in the treatment of cancer can essentially be divided into those that target essential functions and those that target non-essential functions [22]. An example of a drug that targets 'non-essential' functions is the estrogen-receptor modulator, tamoxifen (an antihormonal), which targets specialized tissue (breast epithelium) that is non-essential for life. Traditional cytotoxic pharmaceuticals target essential functions like cell division, which explains why their toxicity is not specific but affects multiple systems in the body. There are other drugs developed more recently that inhibit parts of key signaling pathways such as kinases [22] and are intended to be more specific and less destructive. An example is the drug, Imatinib which is used for chronic myeloid leukemia. Those that target upstream functions in signaling networks have less general toxicity than those that aim farther downstream. However, many of these drugs also affect other crucial survival and proliferation pathways whose destruction is more deleterious [22]. The paradigm for all of these drugs is the assumption that cancer can be treated by targeting and either inhibiting or destroying a single function. As we have seen from the discussion of systems biology, this logic does not fit with what we know about gene-environment interactions and the paradigm has not met with a great deal of success.

Schipper et al. [23] think that we need to shift our perspective towards seeing malignant neoplasms as the result of a process of dysregulation. *The importance of this concept, is that dysregulation is often reversible *[23]. This shifts the paradigm from one that targets the killing of renegade cancer cells [24], to one that focuses on reversing the complex interactions involved in systemic dysregulation [25], Cell behavior (e.g., out of control proliferation) is seen as a result of regulatory imbalance and not as the initiating factor. From a pharmacological perspective, this would require a completely new approach.

Schipper et al., illustrate their point with the example of a scientist using a genetically engineered mouse to investigate the effect of protein A on protein B. The mouse is engineered to knock out either A or B so that its function can be investigated by examining how the system functions without it. The advantage of this model is that it facilitates understanding of the general function of the gene or protein when the dysfunction is structural. However, *it does not mimic the process by which a gene or protein becomes dysfunctional in an intact organism*. Humans are not born lacking entire genes (except in very rare cases). In humans, cancer develops in intact systems, which become dysfunctional through interactions with environmental, immunologic, genetic, viral, epigenetic, behavioral, and other factors. The immune-incompetent animal model supports the theory that immune-incompetence promotes cancer, but it does not mimic the process that leads to tumor initiation and progression in humans. A functional change of A or B (e.g., under expression of a tumor suppressor gene) in an intact organism triggers a response from other systems. Sometimes the response is successful and the dysregulation is corrected (e.g. through tumor suppression, apoptosis, etc.), in others it is not successful and dysfunction continues or worsens. Both rats and humans have natural defense mechanisms against neoplasia, which is why it is so difficult to get a tumor to develop in an immunocompetent animal [26]. The protective systems respond (e.g., to injected cancer cells) by repairing the damage, eliminating the aberrant cells, or suppressing the process of tumor formation. The suggestion made by Schipper et al. that changing the paradigm of clinical oncology from one of tumors as morphological entities to cancer as a process of regulatory imbalance would address some of the failures by changing research strategies. *The fact that cancerous cells can be inserted into an animal and not develop into a tumor, reinforces the theory that it is not the characteristics of the cells themselves, that result in cancer, but the properties emerging from the interaction between the cell and other response systems*.

The receptors, genes, cells and signaling pathways that have been targets for pharmacologic intervention are all part of dynamic networks. These networks are characterized by emergent properties and an adaptability of function that allows them to respond to the needs and demands of the surrounding environment [27,28]. Above all, they are characterized by the fact that they are not isolated but continually interact with each other. This is why drugs that target single functions can have such deleterious 'side' effects. For this reason, the knockout model commonly used in cancer drug development (the nude mouse model) may be a poor model for prediction of response systems that are relevant to cancer etiology and progression [8]. These mice do not mimic what happens in the natural development of cancer because there is no adaptability or flexibility in the part of the system that is genetically engineered. Deletion of function is permanent. This does not mean that we should stop using this model but that in order to get from 'omics' to complex disease phenotypes, we need to complement it with other methods, such as molecular epidemiology, that facilitate the examination of the process of cancerogenesis in humans. The immunodeficiency that allows cancer to grow can take many forms and evolves over many years. Sudden total structural deletion (i.e., knockout) doesn't occur. The body is equipped with multiple DNA repair, immune and tumor suppressor mechanisms that usually destroy aberrant or mutated cells. These systems can become dysfunctional through aging, infection, or multiple environmental onslaughts. When that occurs, host susceptibility to carcinogens increases. Once a tumor is initiated, it changes the interactions between systems, modifying the environment and being modified by it. This perspective on cancer etiology defines cancer as an evolving process of regulatory imbalance. The problem of treatment and prevention then becomes one of how to identify important stages in this process and target an appropriate intervention to reverse the dysregulation as early as possible.

### Epigenetic Mechanisms

The field of epigenetics is providing data that may help us find some answers to these questions. Although tumors are localized at individual sites (e.g., lung, colon, breast), malignancy is characterized by global gene expression changes including genome wide hypomethylation of DNA and chromosomal modification [29], including hypoacetylation of chromatin [30-33]. Along with these global changes, there are site specific epigenetic modifications such as hypermethylation (suppression) of CpG islands in promoter regions that in a healthy system would lead to tumor suppression [29]. *These types of epigenetic changes have a stronger association with tumor progression than do specific mutations *[34], providing additional support for the theory that cancer indeed involves a systemic process. According to a review of genetics and epigenetics in cancer [34], the types of expression changes that are ubiquitous in cancer, namely methylation, loss of imprinting and chromatin modifications, can influence phenotypes through regulation *without underlying changes *(e.g. mutations) *in the sequence of DNA base pairs*. There are multiple mechanisms through which expression changes occur including (but not limited to) the addition of molecules to DNA (methylation), neutralizing of the charge between DNA and histone tails (around which the DNA is wrapped) allowing easy access by transcription factors, post translational modifications and loss of imprinting.

The changes that these modifications reflect are not confined to single functions or signaling pathways but are genome wide. Gene networks are normally quite robust because their nonlinear (scale-free) structure means that the majority of nodes have only one or two links, while a few have many more. This helps to maintain equilibrium by reducing the probability that malfunction in a single gene (node) in the network will knock out the whole system. Therefore, the genome wide expression changes occurring in cancer indicate multiple systemic malfunctions. This pinpoints the difficulty in explaining tumor initiation and progression with single mutations, despite the fact that mutations sometimes serve as markers of tumor progression. The multiple genome wide expression changes that are common to most cancers may contribute to the difficulty in replicating some of the candidate gene studies related to cancer.

The fact that the same candidate gene can be associated with different phenotypes depending on global gene expression patterns lies at the heart of gene-environment interactions. It isn't the gene itself, but its expression (behavior) in the context of other systemic factors that determines risk. Thus, *the same genotype may be associated both with normal and abnormal behavior*. Metastable epialleles are loci that can be epigenetically modified in a manner that is variable and reversible so that a distribution of phenotypes can be produced from genetically identical cells [11]. This is another reason why the effects of genes and environment cannot be understood with additive models. Biologically, genes are up- and down-regulated in a dynamic, ongoing manner based on changing demands and inputs to the system. Since the epidemiologic data on cancer clearly indicate the etiological importance of environmental factors, gene expression and epigenetic mechanisms are receiving increased focus and importance as mediating mechanisms in the characterization of tumor initiation and progression.

The concept of cancer as a process rather than a structural entity is also illustrated by research on cancers associated with well known risk factors (e.g., lung cancer). Smoking is a documented risk factor [35] that it is responsible for about 130,000 deaths annually from active and 22,200 from passive smoking [36]. However, although 80% - 90% of lung cancer patients are current or former tobacco smokers, only 10% - 15% of smokers actually develop lung cancer [37,38], and about 15% of men and 53% of all women with lung cancer worldwide (percentage of women is lower in the U.S. [39]) were never smokers [40]. These epidemiological findings strongly suggest that there are host differences in susceptibility to lung cancer and that the likelihood of a smoker developing lung cancer depends not only on how much and how long s/he has smoked but also on the presence of other etiological risk factors. Such factors include 'at risk' genotypes [41,42], arsenic exposure [43,44], low socioeconomic status [45], and other environmental carcinogens. Population-based studies are an important complement to *in vitro *and animal studies because they identify real-life behavioral and environmental risk factors. However, to be useful, these studies should be powered not only to identify the relevant exposures but to measure change in clinical and gene expression profiles over time. They should include genetic, epigenetic, clinical and other physiological measures that can elucidate the mechanisms and process of cancerogenesis. Nonlinear analytic methods should be utilized to model the interactions.

When modeling gene-environment interactions, it is important to remember that protective factors as well as risk factors cause epigenetic changes. One of the most well known protective factors with respect to cancer is diet. Dietary nutrients such as foliate and vitamin B12 affect the availability of methyl groups for DNA methylation, which is important in carcinogenesis [46-49]. DNA methylation is also inhibited by phytoestrogens such as genistein in soy products [50] and EGCG. The major catechin in green tea inhibits DNA methyltransferase and also reactivates expression of epigenetically silenced genes such as RAR-beta2 [51,52]. It has been demonstrated that treatment of cancer cells with EGCG, can cause the demethylation of CpG islands in promoter regions and the reactivation of methlyation-silenced genes such as p16 [53], which is commonly hypermethylated in lung cancer [54]. The methylation of histones [55] and histone acetylation can be modified by short-chain fatty acids, such as butyrate. These fatty acids act as histone deacetylase inhibitors [56] and can also increase expression of epigenetically silenced genes [57].

The fact that there are usually multiple environmental influences at work simultaneously means that there are effects on multiple interconnected subsystems and signaling pathways and that the influences on each can vary in multiple ways (e.g., they can be synergistic, antagonistic, additive, etc.). This accounts for the enormous complexity and temporal variation characterizing tumor development. It also elucidates why even such a well known risk factor as smoking only sometimes results in cancer and why, despite this toxic exposure, it can take many decades for lung cancer to develop. The body utilizes multiple repair mechanisms to try to contain the damage and is actually capable of stopping or reversing the process if the smoker quits soon enough. If there are protective factors influencing gene expression, this aids in the process.

Schipper et al., conceptualize cancer as a process of regulatory imbalance that is potentially reversible. The data on smoking and lung cancer supports this. The complex process of gene expression changes in non-mutated genes supports the theory that knock-out models are inadequate for understanding the process of oncogenesis. The reason is that individual mutations, DNA adducts or expression changes can be repaired, destroyed or reversed. However, if the burden of risk accumulates to the point of overwhelming the systems' defenses, the result is the breakdown of repair mechanisms and signaling networks that initiate dysfunction. Changes in gene expression can be responsible both for activating oncogenes and for turning off tumor suppressor genes. *These expression changes occur because of influences from the microenvironment in the cell, which reflects and is influenced by factors originating both inside and outside the cell and the body*. It is easy to see that this is a complex process that cannot be adequately described or understood by focusing only on single genes or signaling pathways.

## Presentation of the Hypothesis

The hypothesis of this paper is that real progress in treatment efficacy will not be achieved until we switch from a reductionist paradigm of tumor etiology to a systems model. We must move to a frame of reference that encompasses the complexity involved in tumor initiation and progression in humans and utilize this information to construct multidisciplinary approaches to treatment and prevention that target the reversal of dysfunctional process. This shift in treatment strategy requires increased knowledge of the temporal progression of complex systemic dysregulation associated with specific cancer phenotypes, including how to identify risk factor clusters interacting with specific genotypes, the epigenetic and physiological precursors of tumor initiation, and the steps required to reverse dysregulation. It involves a shift from targeting the identification of genes and signaling pathways involved in tumor progression to the identification of multiple mechanisms that disable DNA repair, apoptosis and immune function, increasing the susceptibility of the host organism to tumorogenesis. The mutations that are being identified in tumor progression may be the result and not the cause of tumor initiation.

### Host Susceptibility

Host susceptibility is the adaptability and responsiveness of multiple physiological systems that facilitate the body's ability to maintain a dynamic equilibrium in the face of risk factor exposure. Host susceptibility to cancerous changes is high when systemic adaptability is low and the ability to respond appropriately to challenge is reduced. Since lung cancer has well known risk factors it is a good example for continuing the discussion of etiologic complexity. The question is why only a minority of smokers develop lung cancer. The data seem to point to a context of phenotypic susceptibility which cannot be reduced to single genes or environmental factors. Given the current state of knowledge, the most logical hypothesis is that there are both genetic and environmental contributors to this vulnerable phenotype that interact in complex ways that are still unclear.

Research on nicotine addiction and lung cancer has investigated genes with both low-penetrance and high-frequency as well as those with high-penetrance and low frequency. A review of this field [58], reports that although a number of loci have shown failure to replicate, there are genomic regions of genetic susceptibility to nicotine dependence on chromosomes 3-7, 9-11, 17, 20 and 22 that have been found to be suggestive or significant in at least two independent samples. This indicates that nicotinic subunit genes are plausible contributors to phenotypic lung cancer vulnerability.

However, we also know that a distribution of phenotypes can be produced from genetically identical cells [11]. Therefore, in addition to genotype, factors influencing gene expression would be an important area of focus with respect to mediating mechanisms. Smoking is the most obvious and it affects multiple systems. It has been demonstrated to up-regulate 23 lipid metabolites, creating a profile that is consistent with down-regulation of alkyl-DHAP in human lung cancer tissues [59]. It also impacts bronchial airway gene expression, heterogeneity of which correlates with smoking-related disease risk [60]. In non-small cell lung cancer, it has been shown that there is a strong link between hypomethylation of Line-1 and Alu transposons that is significantly associated with genomic instability [61], meaning that this epigenetic pattern creates susceptibility for mutation. The examination of gene expression in different lung cancer tumor types (small-cell, adenocarcinoma, squamous-cell carcinoma and non-small cell lung carcinomas) has revealed methylation changes that reflect multiple functional pathways: apoptosis, DNA repair (MGMT), RAS signaling, cell cycle, and invasion [54]. Furthermore, *methylation rates of certain genes (e.g. p16, APC and LCINS) differ between smokers and non-smokers with lung cancer *and *diet influences methylation patterns in smokers *[62], indicating that gene expression profiles are a very good measure of variation in risk [63,64]. So it is becoming clear that smoking is associated with multiple epigenetic changes and that these changes interact with other epigenetic changes caused by environmental factors (e.g., diet) that can influence phenotypic susceptibility for lung cancer.

An additional epigenetic mechanism that is important in lung and other cancers is loss of imprinting [11]. About 1% of human genes are imprinted, which means that their expression is not Mendelian but determined by the parent of origin. Autosomal imprinted genes are structurally diploid (i.e. there is a copy from each parent) but *functionally haploid*, meaning that only one copy is expressed. Loss of imprinting (LOI) of IGF2 is common in lung cancer [11,65]. One study reported that in surgical specimens from lung cancers 6 out of 12 adenocarcinomas, 5 out of 11 squamous cell carcinomas, 2 out of 3 large cell carcinomas and 1 of 4 small cell carcinomas exhibited loss of imprinting [66], demonstrating that LOI is not specific to one type of lung cancer. Similar figures for LOI in adnenocarcinomas - 47% were also found in another study. That study also found LOI for mesoderm-specific tranxript (MEST) [67] and revealed that bi-allelic expression of IGF2 was observed even in the earliest stage tumors. Loss of imprinting of IGF2, otherwise a normal growth promoting gene causes its growth promoting potential to become over expressed and oncogenic.

So how are these mechanisms related to tumor development? It is generally accepted that solid tumors progress through multiple stages, from benign, fairly well-differentiated tumors through stages of genetic instability but low invasiveness, to a stage where they are metastatic and characterized by increasing genetic changes [33]. However, there are no known single genes or genetic mutations that can account for this process, nor are there any known mutations that can account for metastasis or invasion [33]. There are however, multiple gene expression changes that have been demonstrated to contribute to tumor progression. Data suggest that more loss of tumor suppressor gene function may occur through epigenetically mediated gene transcription repression than via frank gene mutations [30,31,68]. This is exemplified by the epigenetic silencing of Hic-1 involved in modulating the activity of the p53 tumor suppressor gene [69,70]. *Importantly, it has been demonstrated that epigenetic change precedes cancer and confers risk for cancer *[65].

### Apoptosis

Cancer as a process is nowhere more evident than in apoptosis or programmed cell death. Apoptosis is one of the body's normal mechanisms for disposing of old or damaged cells. In healthy organisms, the rate of cell turnover is in equilibrium. The rates of cell death and cell proliferation are approximately balanced. In cancer, however, the system of apoptotic signaling becomes dysregulated, allowing abnormal cell proliferation and facilitating tumor formation. Apoptosis can be a reaction to many different types of stimuli, including but not limited to radiation, cigarette smoke, chemical pollutants or DNA damage caused by the body's own metabolic products [71]. These processes involve many systems including micro RNAs [71-75], tumor suppressor genes [76], BH3-only pro-apoptotic proteins as sensors of cell damage [77], the Bcl-2 anti-apoptotic proteins [76,78,79], the TNF receptor [80], mitochondrial processes [81], and many, many more. It goes without saying that epigenetic changes are an important part of this process. What is more important is that these changes are reversible [82].

Meticulous molecular research is being conducted to understand the function of the many parts of this process. The dynamics are so complex that a summary would be beyond the scope of this article. However, apoptosis is an important area for multidisciplinary approaches because it involves key, potentially reversible processes important in the etiology and progression of cancer. To understand the function of individual genes, proteins, and signaling pathways, models such as Drosophila are being used to control for stimuli that would make the data on function ambiguous or difficult to interpret. Complementing these methods with a systems biology approach using data not only from Drosophila but from intact animal models and humans to predict the influence of these systems on the behavior of other systems and verifying this experimentally, constitute important steps in a multi-disciplinary approach. Host susceptibility is an emergent property resulting from the interaction of multiple complex systems. Causality is bi-directional: not only do individual genes and signaling pathways influence what happens to the system as a whole, but organ systems and environmental factors influence activity at the molecular level.

## Allostatic Load

We have used the term, host susceptibility to connote phenotypic risk for cancer. The cumulative health and adaptability of the organism as a whole has also been the focus of extensive research under a parallel concept, 'allostatic load'. The term allostasis is used to describe the dynamic ability of the body to maintain stability through continual change and to modulate new challenge based on prior experience [83]. For example, if there is a frequent need for response from a particular system (e.g., the sympathetic nervous system), it may adjust its basal activity to maintain a higher preparedness or 'vigilance' at rest than at earlier time points when there were fewer demands, because it is readying itself to respond quickly to the next challenge. As long as the demands on the body are reasonable, a state of dynamic equilibrium and healthy functioning is maintained (e.g. blood pressure remains stable, plasma lipids are within reasonable ranges, heart rate variability is fairly high). However, coping resources can become overwhelmed when challenges are too frequent, long lasting or of such magnitude that they overwhelm functional systems. At such times, there is enough flexibility of function in other systems so that back-up efforts can be directed to compensate for the overload. However, the back-up systems pay a price by re-directing their energy from usual tasks to help the system in need. If the need for back-up continues, these systems may eventually not be able to adequately perform primary functions or to mobilize restorative mechanisms by returning to a healthy basal level between challenges. This can set off a chain reaction, requiring other systems to pick up the slack. If the overload continues, feedback within and between systems, will eventually break down. Under these conditions, even during relatively quiescent periods, the body remains mobilized for action. Allostatic load is the price that tissues and organs pay when this over- activity causes the system to become progressively dysregulated so that the feedback mechanisms that normally maintain balance no longer function optimally [84,85]. A 90 year old with multiple co-morbidities is an example of someone with a high allostatic load, while a healthy 20 year old athlete has a low allostatic load. The ability of the 20 year old body to respond with robust defenses to risk factors such as poor diet or smoking is still strong, while that of the 90 year old is vulnerable to even mild challenge. This may be why the prevalence of chronic diseases such as cancer and cardiovascular diseases is rare in younger age groups: allostatic load increases with age.

Lung cancer development is an interesting example of allostatic load because it shows every indication of being a disease of cumulative burden. Smokers develop lung cancer only after decades of exposure. In young healthy individuals, the body mounts strong defenses and is still able to cope with the onslaught of polycyclic aromatic hydrocarbons present in cigarettes. Epidemiologic data indicate that former smokers can have similar risks as never smokers if they stop smoking early enough [86], indicating that the lungs can repair themselves even after years of smoking. This is very consistent with the theory that cancer is a process of dysregulation, which is potentially reversible, rather than a morphological entity that must be destroyed.

### Low Socioeconomic Status Contributes to Allostatic Load

The literature shows that allostatic load is elevated in people with low socioeconomic status (SES) [87] and that low SES is also one of the factors associated with lung cancer [88-90] and with cancer incidence and mortality in general [91-95].

Even though epidemiologic evidence shows that smoking is extremely important, it also indicates that smoking is not the only explanation for SES disparities. A prospective study of 22,387 men and women in Sweden reported that low SES was a significant predictor of lung cancer *even after controlling for smoking*, *inhalation habits*, age and marital status [45]. Since Sweden has single payer national health care, disparities in access to care are not a likely explanation. Genetic factors undoubtedly play an important role in host susceptibility as indicated earlier, however they are less likely to account for SES disparities in lung cancer. Although it has been shown that low SES is associated with higher allostatic load as well as disparities in cancer morbidity and mortality, the question of mechanism is still unclear.

Research on 1552 female twins may supply some helpful information. It has been shown that low SES is associated with shorter telomere length (regions of repetitive DNA fragments at the end of chromosomes that protect them from replication failures) in white blood cells [96]. A pathway from DNA replication failures to mutations might be hypothesized but still begs the question concerning the association between low SES and shorter telomere length. Is there a true association or is this an artifact caused by a confounder?

Data suggest that the association may be real. It has been found that low telomere length and low telomerase activity in leukocytes are associated with exaggerated autonomic reactivity (catecholamines and cortisol) to acute mental stress and elevated nocturnal epinephrine [97], showing that short telomere regions are associated with increased secretion of stress hormones. An association between SES and stress has also been found in children with asthma. This is interesting because asthma is one of the areas where unpredictable transitions from clinically stable to unstable states occur. It has been shown that low SES children exhibit over expression of genes regulating stress responses and inflammatory processes compared with asthmatic children from high SES environments [82]. The low SES children in that study exhibited heightened production of IL-5 and IL-13, higher eosinophil counts and higher chronic and perceived stress [98]. Thus, low SES has been associated with psychological stress and a multilevel systemic response ranging from gene expression changes to immune function. Immune function is important in cancer and tumor infiltrating lymphocytes are considered to represent an immune response against tumor antigens [99]. There are also indications that lymphocyte distribution in lymph nodes is a significant prognostic biomarker in cancer [99].

In sum, the data indicate that the association between lung cancer and low SES is partially mediated by smoking but that a significant amount of the variance remains unexplained after adjusting for the effects of smoking. Data also indicate that low SES is associated with other important factors that may be contributing to host susceptibility. Stress and the physiological response cascade associated with it, is a strong candidate but the association is not straightforward. The dynamics of stress physiology are not linear. This is illustrated by differences in immune response to acute and chronic stress. Antigen-specific, cell-mediated immune responses known as delayed type hypersensitivity are enhanced in acute stress but attenuated in chronic stress [100]. This nonlinear response is not surprising since it fits the concept of allostatic load and is typical of a system that responds powerfully to an initial stimulus but tries to reserve energy by responding with lower magnitude reactions to chronic, repeated challenge. What these data indicate is that there are a whole host of risk factors that are important in the process of dysregulation.

## Testing and Implications of the Hypothesis

The fact that cancerous cells can be inserted into an animal and not develop into a tumor, reinforces the theory that it is not the characteristics of the cells themselves, but the properties emerging from the interaction between cells and other response systems that results in cancer. Because many genes have multiple functions depending on the way they are expressed, the relative effect of individual genes is dependent on the state of the surrounding systems and varies within defined limits as the state changes. The same principle applies to environmental risk factors. The effect or lack of effect related to single factors is dependent on which other factors are simultaneously present, the timing of exposure, and the robustness of immune, DNA repair, and apoptotic defenses. Thus, treatments limited to single components of this system (e.g. genes, hormone receptors, signaling pathways) have a limited probability of success.

So what is the solution? The primary focus of current oncology research is the identification of SNPs or genetic mutations that predict progression in already existing tumors, or identifying genes and signaling pathways associated with tumor initiation in immune-compromised animals. Since it has become increasingly clear that cancer is a dynamical process of dysregulation which increases host susceptibility to tumorigenesis, a better strategy would be to *focus on systemic precursors of cancer in non-diseased organisms*. This would facilitate characterization of mechanisms that derail the abundant natural defenses normally in place to prevent tumors. This approach requires multi-disciplinary research that encompasses a combination of basic science, animal models that also include non- immune-compromised animals, and human studies, including prospective epidemiologic research in 'at risk' populations to characterize genetic and environmental risks, interactions and epigenetic mechanisms. Nonlinear mathematics and systems modeling are necessary to assure proper analysis and interpretation of the data.

In conclusion, significantly improving treatment outcomes will require a shift away from treatments that 'seek and destroy' aberrant cells, tumors, or signaling pathways to one that tailors interventions to the complex temporal sequence of gene-environment interactions involved in the process of tumor initiation and progression. Above all, it will require a paradigm shift, moving from the goal of eliminating malignant cells, to a model that focuses on intervening at a time point or stage in the process where dysfunction can be reversed.

## Competing interests

The authors declare that they have no competing interests.

## References

[B1] Pedersen NL De Raad B, Hofstee WKB, Van Heck GL The nature and nurture of personality Personality Psychology in Europe 1994 Tilburg: Tilburg University Press 110 132

[B2] "Epigenetics" means what we eat, how we live and love, alters how our genes behave http://www.dukehealth.org/health_library/news/9322

[B3] Kruse J Gu W Modes of p53 regulation Cell 2009 137 4 609 622 10.1016/j.cell.2009.04.050 19450511 PMC3737742

[B4] Riley T Sontag E Chen P Levine A Transcriptional control of human p53-regulated genes Nature Reviews Molecular Cell Biology 2008 9 5 402 412 10.1038/nrm2395 18431400

[B5] Musani S Shriner D Liu N Feng R Coffey C Yi N Tiwari H Allison D Detection of gene× gene interactions in genome-wide association studies of human population data Hum Hered 2007 63 2 67 84 10.1159/000099179 17283436

[B6] Gardner M Bertranpetit J Comas D Worldwide genetic variation in dopamine and serotonin pathway genes: implications for association studies American journal of medical genetics Part B, Neuropsychiatric genetics: the official publication of the International Society of Psychiatric Genetics 2008 147 7 1070 10.1002/ajmg.b.30717 18270970

[B7] Testa B Kier L Carrupt P A systems approach to molecular structure, intermolecular recognition, and emergence-dissolvence in medicinal research Medicinal Research Reviews 1997 17 4 10.1002/(SICI)1098-1128(199707)17:4<303::AID-MED1>3.0.CO;2-# 9211395

[B8] Alberghina L Höfer T Vanoni M Molecular networks and system-level properties Journal of Biotechnology 2009 144 3 224 33 10.1016/j.jbiotec.2009.07.009 19616593

[B9] Poirier MG Oh E Tims HS Widom J Dynamics and function of compact nucleosome arrays Nature Structural & Molecular Biology 2009 16 9 938 944 10.1038/nsmb.1650 19701201 PMC2748796

[B10] Agutter P Wheatley D About Life: Concepts in Modern Biology 2007 Dordrecht, The Netherlands: Springer

[B11] Jirtle R Skinner M Environmental epigenomics and disease susceptibility Nature reviews genetics 2007 8 4 253 262 10.1038/nrg2045 17363974 PMC5940010

[B12] Voss A Schroeder R Truebner S Goernig M Figulla H Schirdewan A Comparison of nonlinear methods symbolic dynamics, detrended fluctuation, and Poincare plot analysis in risk stratification in patients with dilated cardiomyopathy Chaos: An Interdisciplinary Journal of Nonlinear Science 2007 17 015120 10.1063/1.2404633 17411277

[B13] Peng C Buldyrev S Hausdorff J Havlin S Mietus J Simons M Stanley H Goldberger A Non-equilibrium dynamics as an indispensable characteristic of a healthy biological system Integrative Psychological and Behavioral Science 1994 29 3 283 293 10.1007/BF02691332 7811648

[B14] Stanley H Buldyrev S Goldberger A Havlin S Peng C Simons M Scaling features of noncoding DNA Physica A 1999 273 1-2 1 18 10.1016/S0378-4371(99)00407-0 11542924

[B15] Goldberger A Amaral L Hausdorff J Ivanov P Peng C Stanley H Fractal dynamics in physiology: alterations with disease and aging National Acad Sciences 2002 99 2466 2472 10.1073/pnas.012579499 PMC128562 11875196

[B16] Scheffer M Bascompte J Brock WA Brovkin V Carpenter SR Dakos V Held H van Nes EH Rietkerk M Sugihara G Early-warning signals for critical transitions Nature 2009 461 7260 53 10.1038/nature08227 19727193

[B17] Kirschner M The meaning of systems biology Cell 2005 121 4 503 504 10.1016/j.cell.2005.05.005 15907462

[B18] Ahn AC Tewari M Chi-Sang P Phillips RS The Limits of Reductionism in Medicine: Could Systems Biology Offer an Alternative? PLoS Medicine 2006 3 5 e208 0713 10.1371/journal.pmed.0030208 16681415 PMC1459480

[B19] Grigorov M Global dynamics of biological systems from time-resolved omics experiments 2006 22 Oxford Univ Press 1424 1430 16585068 10.1093/bioinformatics/btl119

[B20] Dollery CKR Challis R Delpy D Edwards D Henney A Kirkwood T Noble D Rowland M Tarassenko L Williams D Smith L Systems biology: A vision for engineering and medicine A Report from the Academy of Medical Sciences and the Royal Academy of Engineering 2007

[B21] Vera J Wolkenhauer O A systems biology approach to understand functional activity of cell communications systems Methods in Cell Biology 2008 90 399 415 full_text 19195559 10.1016/S0091-679X(08)00817-0

[B22] Kamb A Wee S Lengauer C Why is cancer drug discovery so difficult? Nature Reviews Drug Discovery 2006 6 2 115 120 10.1038/nrd2155 17159925

[B23] Schipper H Goh C Wang T Shifting the cancer paradigm: must we kill to cure? Journal of Clinical Oncology 1995 13 4 801 7707104 10.1200/JCO.1995.13.4.801

[B24] Dolgin E Cancer metastasis scrutinized Nature 2009 461 7266 854 855 10.1038/461854b 19829337

[B25] Hoeijmakers J DNA Damage, Aging, and Cancer New England Journal of Medicine 2009 361 15 1475 10.1056/NEJMra0804615 19812404

[B26] Shankaran V Ikeda H Bruce AT White JM Swanson PE Old LJ Schreiber RD IFNgamma and lymphocytes prevent primary tumour development and shape tumour immunogenicity Nature 2001 410 6832 1107 10.1038/35074122 11323675

[B27] Jeong H Tombor B Albert R Oltvai Z Barabasi A The large-scale organization of metabolic networks Nature 2000 407 6804 651 654 10.1038/35036627 11034217

[B28] Chauvet G Hierarchical functional organization of formal biological systems: A dynamical approach. I. The increase of complexity by self-association increases the domain of stability of a biological system Philosophical Transactions: Biological Sciences 1993 339 1290 425 444 10.1098/rstb.1993.0040 8098872

[B29] Callinan PA Feinberg AP The emerging science of epigenomics Human Molecular Genetics 2006 15 Spec No 1 R95 101 10.1093/hmg/ddl095 16651376

[B30] Baylin SB Chen WY Aberrant Gene Silencing in Tumor Progression: Implications for Control of Cancer Cold Spring Harbor Symposia on Quantitative Biology 2005 70 427 433 10.1101/sqb.2005.70.010 16869780

[B31] Jones P Baylin S The fundamental role of epigenetic events in cancer Nature reviews genetics 2002 3 6 415 428 12042769 10.1038/nrg816

[B32] Jones P Baylin S The epigenomics of cancer Cell 2007 128 4 683 692 10.1016/j.cell.2007.01.029 17320506 PMC3894624

[B33] Feinberg A Ohlsson R Henikoff S The epigenetic progenitor origin of human cancer Nature reviews genetics 2006 7 1 21 33 10.1038/nrg1748 16369569

[B34] Bjornsson HT Daniele Fallin M Feinberg AP An integrated epigenetic and genetic approach to common human disease Trends in Genetics 2004 20 8 350 358 10.1016/j.tig.2004.06.009 15262407

[B35] Burns D Tobacco-related diseases 2003 Elsevier 244 249 14702858 10.1053/j.soncn.2003.08.001

[B36] Gan Q Smith K Hammond S Hu T Disease burden of adult lung cancer and ischaemic heart disease from passive tobacco smoking in China British Medical Journal 2007 16 6 417 10.1136/tc.2007.021477 PMC2807198 18048620

[B37] Alberg AJ Samet JM Epidemiology of Lung Cancer CHEST 2003 123 21S 10.1378/chest.123.1_suppl.21S 12527563

[B38] Mattson M Pollack E Cullen J What are the odds that smoking will kill you? Am Public Health Assoc 1987 77 425 431 10.2105/AJPH.77.4.425 PMC1646951 3826460

[B39] Subramanian J Govindan R Lung cancer in never smokers: a review Journal of Clinical Oncology 2007 25 5 561 10.1200/JCO.2006.06.8015 17290066

[B40] Parkin D Bray F Ferlay J Pisani P Global cancer statistics, 2002 CA: a cancer journal for clinicians 2005 55 2 74 108 10.3322/canjclin.55.2.74 15761078

[B41] Sellers T Elston R Stewart C Rothschild H Vogler G Familial risk of cancer among randomly selected cancer probands Genetic epidemiology 1988 5 6 10.1002/gepi.1370050603 3209052

[B42] Tokuhata G Lilienfeld A Familial aggregation of lung cancer in humans Journal of the National Cancer Institute 1963 30 289 13985327

[B43] Guo H Arsenic level in drinking water and mortality of lung cancer (Taiwan) Cancer Causes and Control 2004 15 2 171 177 10.1023/B:CACO.0000019503.02851.b0 15017129

[B44] Hertz-Picciotto I Smith A Observations on the dose-response curve for arsenic exposure and lung cancer Scandinavian journal of work, environment & health 1993 19 4 217 226 8235510 10.5271/sjweh.1480

[B45] Ekberg-Aronsson M Nilsson P Nilsson J Pehrsson K Löfdahl C Socio-economic status and lung cancer risk including histologic subtyping--a longitudinal study Lung cancer 2006 51 1 21 29 10.1016/j.lungcan.2005.08.014 16337709

[B46] Ross S Diet and DNA methylation interactions in cancer prevention Annals of the New York Academy of Sciences 2003 983 197 207 10.1111/j.1749-6632.2003.tb05974.x 12724224

[B47] Choi S Mason J Folate status: effects on pathways of colorectal carcinogenesis The Journal of nutrition 2002 132 8 Suppl 2413S 12163703 10.1093/jn/132.8.2413S

[B48] Harnack L Jacobs D Jr Nicodemus K Lazovich D Anderson K Folsom A Relationship of folate, vitamin B-6, vitamin B-12, and methionine intake to incidence of colorectal cancers Nutrition and cancer 2002 43 2 152 10.1207/S15327914NC432_5 12588695

[B49] Brunaud L Alberto JM Ayav A Gerard P Namour F Antunes L Braun M Bronowicki JP Bresler L Gueant JL Effects of vitamin B12 and folate deficiencies on DNA methylation and carcinogenesis in rat liver Clinical Chemistry & Laboratory Medicine 2003 41 8 1012 1019 10.1515/CCLM.2003.155 12964806

[B50] Lyn-Cook B Blann E Payne P Bo J Sheehan D Medlock K Methylation profile and amplification of proto-oncogenes in rat pancreas induced with phytoestrogens SEBM 1995 208 116 119 10.3181/00379727-208-43842 7534422

[B51] Fang MZ Wang YM Ai N Hou Z Sun Y Lu H Welsh W Yang CS Tea polyphenol (-)-epigallocatechin-3-gallate inhibits DNA methyltransferase and reactivates methylation-silenced genes in cancer cell lines Cancer Research 2003 63 22 7563 7570 14633667

[B52] Fang MZ Chen D Sun Y Christman JK Yang CS Reversal of hypermethylation and reactivation of p16-INK4a, RAR, and MGMT genes by genistein and other isoflavones from soy Clin Cancer Res 2005 11 7033 7041 10.1158/1078-0432.CCR-05-0406 16203797

[B53] Yang CS Fang M Lambert JD Yan P Huang THM Reversal of hypermethylation and reactivation of genes by dietary polyphenolic compounds Nutrition Reviews 2008 66 S18 10.1111/j.1753-4887.2008.00059.x 18673481 PMC2829855

[B54] Belinsky SA Gene-promoter hypermethylation as a biomarker in lung cancer Nature Reviews Cancer 2004 4 9 707 717 10.1038/nrc1432 15343277

[B55] Rea S Eisenhaber F O'Carroll D Strahl B Sun Z Schmid M Opravil S Mechtler K Ponting C Allis C Regulation of chromatin structure by site-specific histone H3 methyltransferases Nature 2000 406 6796 593 599 10.1038/35020506 10949293

[B56] Candido E Reeves R Davie J Sodium butyrate inhibits histone deacetylation in cultured cells Cell 1978 14 1 105 113 10.1016/0092-8674(78)90305-7 667927

[B57] Demary K Wong L Spanjaard RA Effects of retinoic acid and sodium butyrate on gene expression, histone acetylation and inhibition of proliferation of melanoma cells Cancer Letters 2001 163 1 103 107 10.1016/S0304-3835(00)00676-5 11163113

[B58] Li M Identifying susceptibility loci for nicotine dependence: 2008 update based on recent genome-wide linkage analyses Human Genetics 2008 123 2 119 131 10.1007/s00439-008-0473-0 18205015

[B59] Wang-Sattler R Yu Y Mittelstrass K Lattka E Altmaier E Gieger C Ladwig K Dahmen N Weinberger K Hao P Metabolic Profiling Reveals Distinct Variations Linked to Nicotine Consumption in Humans--First Results from the KORA Study PLoS One 2008 3 12 10.1371/journal.pone.0003863 19057651 PMC2588343

[B60] Schembri F Sridhar S Perdomo C Gustafson A Zhang X Ergun A Lu J Liu G Bowers J MicroRNAs as modulators of smoking-induced gene expression changes in human airway epithelium Proceedings of the National Academy of Sciences 2009 106 7 2319 10.1073/pnas.0806383106 PMC2650144 19168627

[B61] Daskalos A Nikolaidis G Xinarianos G Savvari P Cassidy A Zakopoulou R Kotsinas A Gorgoulis V Field JK Liloglou T Hypomethylation of retrotransposable elements correlates with genomic instability in non-small cell lung cancer International Journal Of Cancer Journal International Du Cancer 2009 124 1 81 87 10.1002/ijc.23849 18823011

[B62] Sidley C Picchi M Leng S Willink R Crowell R Flores K Kang H Byers T Gilliland F Belinsky S Multi-vitamins, folate, and Green vegetables protect against gene promoter methylation in the aerodigestive tract of smokers Cancer Research 2010 70 2 568 574 10.1158/0008-5472.CAN-09-3410 20068159 PMC3076796

[B63] Divine KK Pulling LC Marron-Terada PG Liechty KC Kang T Schwartz AG Bocklage TJ Coons TA Gilliland FD Belinsky SA Multiplicity of abnormal promoter methylation in lung adenocarcinomas from smokers and never smokers International Journal Of Cancer Journal International Du Cancer 2005 114 3 400 405 10.1002/ijc.20761 15578700

[B64] Zöchbauer-Müller S Fong K Virmani A Geradts J Gazdar A Minna J Aberrant promoter methylation of multiple genes in non-small cell lung cancers AACR 2001 61 249 255 11196170

[B65] Feinberg A Phenotypic plasticity and the epigenetics of human disease Nature 2007 447 7143 433 440 10.1038/nature05919 17522677

[B66] Suzuki H Ueda R Takahashi T Altered imprinting in lung cancer Nature Genetics 1994 6 4 332 333 10.1038/ng0494-332 8054970

[B67] Kohda M Hoshiya H Katoh M Tanaka I Masuda R Takemura T Fujiwara M Oshimura M Frequent loss of imprinting of IGF2 and MEST in lung adenocarcinoma Molecular Carcinogenesis 2001 31 4 10.1002/mc.1053 11536368

[B68] Herman J Baylin S Gene silencing in cancer in association with promoter hypermethylation New England Journal of Medicine 2003 349 21 2042 2054 10.1056/NEJMra023075 14627790

[B69] Chen WY Wang DH Yen RC Luo J Gu W Baylin SB Tumor Suppressor HIC1 Directly Regulates SIRT1 to Modulate p53-Dependent DNA-Damage Responses Cell 2005 123 3 437 448 10.1016/j.cell.2005.08.011 16269335

[B70] Chen WY Baylin SB Inactivation of tumor suppressor genes: choice between genetic and epigenetic routes Cell Cycle (Georgetown, Tex) 2005 4 1 10 12 15611624 10.4161/cc.4.1.1361

[B71] Bartek J Lukas J The Stress of Finding NEMO Science 2006 311 5764 1110 1111 10.1126/science.1124540 16497923

[B72] Garzon R Calin GA Croce CM MicroRNAs in Cancer Annual Review Of Medicine 2009 60 167 179 10.1146/annurev.med.59.053006.104707 19630570

[B73] Drakaki A Iliopoulos D MicroRNA Gene Networks in Oncogenesis 2009 10 1 35 41 19721809 10.2174/138920209787581299 PMC2699834

[B74] Vasilatou D Papageorgiou S Pappa V Papageorgiou E Dervenoulas J The role of microRNAs in normal and malignant hematopoiesis European Journal of Haematology 2009 9999 9999 10.1111/j.1600-0609.2009.01348.x 19744129

[B75] Mott J MicroRNAs involved in tumor suppressor and oncogene pathways; implications for hepatobiliary neoplasia Hepatology 2009 50 2 630 7 10.1002/hep.23010 19585622 PMC2721015

[B76] Cotter T Apoptosis and cancer: the genesis of a research field Nature Reviews Cancer 2009 9 7 501 507 10.1038/nrc2663 19550425

[B77] Danial NN BAD: undertaker by night, candyman by day Oncogene 2008 27 Suppl 1 S53 70 10.1038/onc.2009.44 19641507

[B78] Tse C Shoemaker A Adickes J Anderson M Chen J Jin S Johnson E Marsh K Mitten M Nimmer P ABT-263: a potent and orally bioavailable Bcl-2 family inhibitor Cancer Research 2008 68 9 3421 10.1158/0008-5472.CAN-07-5836 18451170

[B79] Shoemaker A Mitten M Adickes J Ackler S Refici M Ferguson D Oleksijew A O'Connor J Wang B Frost D Activity of the Bcl-2 family inhibitor ABT-263 in a panel of small cell lung cancer xenograft models Clinical Cancer Research 2008 14 11 3268 10.1158/1078-0432.CCR-07-4622 18519752

[B80] Garofalo M Condorelli G Croce C Condorelli G MicroRNAs as regulators of death receptors signaling Cell Death & Differentiation 2009 17 2 200 8 10.1038/cdd.2009.105 19644509

[B81] Galluzzi LME Kepp O Vitalio Rigoni A Vacchelli E Mitchondrial gateways to cancer MOlecular Aspects of Medicine 2009 31 1 1 20 10.1016/j.mam.2009.08.002 19698742

[B82] Chen SS RavaI A Johnson AJ Hertlein E Te-Hui L Jin VX Sherman MH Shu-Jun L Dawson DW Williams KE Epigenetic changes during disease progression in a murine model of human chronic lymphocytic leukemia Proceedings of the National Academy of Sciences of the United States of America 2009 106 32 13433 13438 10.1073/pnas.0906455106 19666576 PMC2726368

[B83] McEwen B Interacting mediators of allostasis and allostatic load: towards an understanding of resilience in aging Metabolism 2003 52 10 16 10.1053/S0026-0495(03)00295-6 14577057

[B84] McEwen B Stellar E Stress and the individual: mechanisms leading to disease Archives of Internal Medicine 1993 153 18 2093 10.1001/archinte.153.18.2093 8379800

[B85] McEwen B Protective and damaging effects of stress mediators New England Journal of Medicine 1998 338 3 171 179 10.1056/NEJM199801153380307 9428819

[B86] Bjartveit K Tverdal A Health consequences of sustained smoking cessation British Medical Journal 2009 18 3 197 10.1136/tc.2008.026898 19228666

[B87] Szanton S Gill J Allen J Allostatic Load: A Mechanism of Socioeconomic Health Disparities? Biological Research for Nursing 2005 7 1 7 10.1177/1099800405278216 15919999 PMC2874580

[B88] Geyer S Social inequalities in the incidence and case fatality of cancers of the lung, the stomach, the bowels, and the breast Cancer Causes and Control 2008 19 9 965 974 18431680 10.1007/s10552-008-9162-5

[B89] Chu KC Miller BA Springfield SA Measures of racial/ethnic health disparities in cancer mortality rates and the influence of socioeconomic status Journal of the National Medical Association 2007 99 10 1092 1104 17987912 PMC2574395

[B90] Lewis DR Clegg LX Johnson NJ Lung disease mortality in the United States: the National Longitudinal Mortality Study The International Journal Of Tuberculosis And Lung Disease: The Official Journal Of The International Union Against Tuberculosis And Lung Disease 2009 13 8 1008 1014 19723382 PMC2765862

[B91] Hausauer AK Keegan T Chang ET Glaser SL Howe H Clarke CA Recent trends in breast cancer incidence in US white women bycounty-level urban/rural and poverty status BMC Medicine 2009 7 31 42 10.1186/1741-7015-7-31 19558637 PMC2714853

[B92] Warnakulasuriya S Significant oral cancer risk associated with low socioeconomic status Evidence-Based Dentistry 2009 10 1 4 5 10.1038/sj.ebd.6400623 19322216

[B93] Frederiksen B Osler M Harling H Ladelund S Jørgensen T The impact of socioeconomic factors on 30-day mortality following elective colorectal cancer surgery: A nationwide study European Journal of Cancer 2009 45 7 1248 1256 10.1016/j.ejca.2008.11.035 19136251

[B94] Siegel RL Jemal A Thun MJ Hao Y Ward EM Trends in the incidence of colorectal cancer in relation to county-level poverty among blacks and whites Journal of the National Medical Association 2008 100 12 1441 1444 19110912 10.1016/s0027-9684(15)31544-3

[B95] Ou S-HI Ziogas A Zell JA Prognostic factors for survival in extensive stage small cell lung cancer (ED-SCLC): the importance of smoking history, socioeconomic and marital statuses, and ethnicity Journal Of Thoracic Oncology: Official Publication Of The International Association For The Study Of Lung Cancer 2009 4 1 37 43 19096304 10.1097/JTO.0b013e31819140fb

[B96] Cherkas LF Aviv A Valdes AM Hunkin JL Gardner JP Surdulescu GL Kimura M Spector TD The effects of social status on biological aging as measured by white-blood-cell telomere length Aging Cell 2006 5 5 361 365 10.1111/j.1474-9726.2006.00222.x 16856882

[B97] Epel ES Lin J Wilhelm FH Wolkowitz OM Cawthon R Adler NE Dolbier C Mendes WB Blackburn EH Cell aging in relation to stress arousal and cardiovascular disease risk factors Psychoneuroendocrinology 2006 31 3 277 287 10.1016/j.psyneuen.2005.08.011 16298085

[B98] Chen E Hanson MD Paterson LQ Griffin MJ Walker HA Miller GE Socioeconomic status and inflammatory processes in childhood asthma: the role of psychological stress The Journal Of Allergy And Clinical Immunology 2006 117 5 1014 1020 10.1016/j.jaci.2006.01.036 16675327

[B99] Pretscher D Distel L Grabenbauer G Wittlinger M Buettner M Niedobitek G Distribution of immune cells in head and neck cancer: CD8+ T-cells and CD20+ B-cells in metastatic lymph nodes are associated with favourable outcome in patients with oro-and hypopharyngeal carcinoma BMC cancer 2009 9 1 292 10.1186/1471-2407-9-292 19698134 PMC2739224

[B100] Dhabhar F Mcewen B Acute Stress Enhances while Chronic Stress Suppresses Cell-Mediated Immunityin Vivo: A Potential Role for Leukocyte Trafficking Brain Behavior and immunity 1997 11 4 286 306 10.1006/brbi.1997.0508 9512816

